# Well-being approaches targeted to improve child and youth health post-COVID-19 pandemic: a scoping review

**DOI:** 10.1186/s12913-024-11140-7

**Published:** 2024-06-21

**Authors:** Stephana Julia Moss, Cynthia Sriskandarajah, Rebecca Brundin-Mather, Michal S. Cherak, Sara J. Mizen, Maia Stelfox, Donna Halperin, Scott Halperin, Sofia B. Ahmed, Diane L. Lorenzetti, Stacie Smith, Micaela Harley, Perri R. Tutelman, Kathryn A. Birnie, Melanie C. Anglin, Henry T. Stelfox, Kirsten M. Fiest, Nicole Racine, Jeanna Parsons Leigh

**Affiliations:** 1 School of Health Administration, Faculty of Health, Dalhousie University, Halifax, NS Canada; 2 Department of Critical Care Medicine, Cumming School of Medicine, University of Calgary, Calgary, AB Canada; 3 Rankin School of Nursing, St. Francis Xavier University, Antigonish, NS Canada; 4 Department of Pediatrics, Faculty of Medicine, Dalhousie University, Halifax, NS Canada; 5 Department of Medicine, Cumming School of Medicine, University of Calgary, Calgary, AB Canada; 6 Department of Community Health Sciences, Cumming School of Medicine, University of Calgary, Calgary, AB Canada; 7 Young Canadians Roundtable on Health, Toronto, ON Canada; 8 Royal’s Institute of Mental Health Research, Ottawa, ON Canada; 9 Department of Oncology, Cumming School of Medicine, University of Calgary, Calgary, AB Canada; 10 Departments of Anesthesiology and Perioperative and Pain Medicine, Cumming School of Medicine, University of Calgary, Calgary, AB Canada; 11 O’Brien Institute of Public Health, Cumming School of Medicine, University of Calgary, Calgary, AB Canada; 12 Chair in Child and Youth Mental Health, University of Ottawa, Ottawa, ON Canada

**Keywords:** COVID-19, Children, Youth, Well-being, Scoping review

## Abstract

**Background:**

Our previous work synthesized published studies on well-being interventions during COVID-19. As we move into a post-COVID-19 pandemic period there is a need to comprehensively review published strategies, approaches, and interventions to improve child and youth well-being beyond deleterious impacts experienced during COVID-19.

**Methods:**

Seven databases were searched from inception to January 2023. Studies were included if they: (1) presented original data on an approach (i.e., *approach applied*) or (2) provided recommendations to inform development of a future approach (i.e., *approach suggested*), (3) targeted to mitigate negative impacts of COVID-19 on child and youth (≤18 year) well-being, and (4) published on or after December 2019.

**Results:**

39 studies (*n* = 4/39, 10.3% randomized controlled trials) from 2021 to 2023 were included. Twenty-two studies *applied* an *approach* (*n* = 22/39, 56.4%) whereas seventeen studies (*n* = 17/39, 43.6%) *suggested* an *approach*; youth aged 13–18 year (*n* = 27/39, 69.2%) were most frequently studied. *Approach applied* records most frequently adopted an experimental design (*n* = 11/22, 50.0%), whereas *approach suggested* records most frequently adopted a cross-sectional design (*n* = 13/22, 59.1%). The most frequently reported outcomes related to good health and optimum nutrition (*n* = 28/39, 71.8%), followed by connectedness (*n* = 22/39, 56.4%), learning, competence, education, skills, and employability (*n* = 18/39, 46.1%), and agency and resilience (*n* = 16/39, 41.0%).

**Conclusions:**

The rapid onset and unpredictability of COVID-19 precluded meaningful engagement of children and youth in strategy development despite widespread recognition that early engagement can enhance usefulness and acceptability of interventions. Published or recommended strategies were most frequently targeted to improve connectedness, belonging, and socialization among children and youth.

**Supplementary Information:**

The online version contains supplementary material available at 10.1186/s12913-024-11140-7.

## Background

After the World Health Organization (WHO) declared the SARS-CoV-2 (COVID-19) disease outbreak a global pandemic, governments worldwide enacted wide-scale policies to prevent the spread of the virus. Public institutions (e.g., schools, libraries) closed and citizens were mandated to wear facial masks and to follow physical distancing protocols. As schools locked down, children and youth were isolated from friends, teachers, and their other community networks; academic environments were drastically changed as extracurriculars and in-person learning supports ceased or were significantly reduced. This social isolation during an unprecedented and uncertain period had a significant negative impact on children and youth [1–4]. Previous research has demonstrated that adverse childhood experiences, such as familial instability due to parental separation or household experiencing substance use or mental health problems, have potentially damaging effects on development and overall health [5].

The literature has established profound impacts from the COVID-19 pandemic on child, youth, and familial well-being (e.g., social, mental, physical, intellectual health) [1] that varies among families and is dependent on economic status, living conditions, race, and other socio-economic factors [2, 3]. Mental health worsened for children in about 1 in 10 families [3] with increased prevalence of fear, depressive and anxiety symptoms, and other negative mental states [4]. For most children and youth, physical inactivity increased, which is associated with increased obesity, anxiety, and depression among youth [6]. Academic learning has fallen behind amidst school closures for most children and youth, and the educational gap between higher and lower income families widened during the pandemic [7–9]. However, although the COVID-19 pandemic posed significant social, educational, and mental health challenges for children and youth, some children and youth experienced only a handful of challenges or evaded these challenges completely [10]. Understanding which children and youth do well in the face of adversity, and why, is equally important to studying difficult experiences among children and youth during the COVID-19 pandemic [11].

Our previous work has synthesized published studies on well-being interventions during the COVID-19 pandemic [12]. As we move into a post-COVID-19 pandemic period—the epoch that arises after the global health and socioeconomic crisis of the COVID-19 pandemic [13]—there is a need to comprehensively review published well-being strategies, approaches, or interventions targeted to improve child and youth well-being beyond the deleterious impacts of the COVID-19 pandemic. The definition for well-being follows the framework from the United Nations H6 + Technical Working Group on Adolescent and Well-being initiative, such that optimal well-being is when children and youth have the support, confidence, and resources to thrive in the context of secure and healthy relationships, realizing their full potential and rights [14].

## Methods

This scoping review aimed to answer the primary research question: What well-being strategies, approaches, or interventions have been developed and tested to mitigate potentially deleterious impacts of the COVID-19 pandemic on child and youth health in the post-COVID-19 pandemic period? We also aimed to answer the secondary research question: What evidence has been published that is intended to directly inform future well-being strategies, approaches, or interventions to mitigate potentially deleterious impacts of the COVID-19 pandemic on child and youth health in the post-COVID-19 pandemic period?

The protocol for this scoping review has been published [15]. This scoping review followed the Arksey O’Malley five-stage scoping review method [16–18], the Scoping Review Methods Manual by the Joanna Briggs Institute [19] and the Preferred Reporting Items for Systematic Review and Meta-Analysis (PRISMA) guideline. The PRISMA extension for Scoping Reviews (PRISMA-ScR) was used to report the findings for the completed scoping review [20].

### Eligibility criteria

Records of primary research studies published on or after December 2019 that presented new data on a well-being strategy, approach, or intervention (i.e., hereafter, *approach applied*) or provided recommendations to inform development of a future well-being strategy, approach, or intervention (i.e., hereafter, *approach suggested*) to mitigate negative impacts of COVID-19 on child and youth (≤18 years) health in the post-COVID-19 pandemic period were considered for inclusion.

The components of population, exposure, comparator, outcome, study design and timeframe are:

- *Population*: Children or youth (≤ 18 years who may or may not have been infected with COVID-19 previously) and their (immediate and extended) families (if presented).
- *Exposure*: Any well-being strategies, approaches, or interventions, including clinical, social, policy or political (specific to children and youth) components to mitigate potentially deleterious impacts (e.g., psychological, physical) of the COVID-19 pandemic on child and youth health as we enter into a post-COVID-19 pandemic period.
- *Comparator*: Any or no comparator were accepted.
- *Outcomes*: Any health outcome operationalized according to published domains (health and nutrition, connectedness, safety and support, learning and competence, and agency and resilience) in the framework from the United Nations H6 + Technical Working Group on Adolescent and Well-being initiative. Studies that provided recommendations to inform development of a future well-being strategy, approach, or intervention needed to have specified the targeted health outcome(s).
- *Study design*: Any empirical or non-empirical study, excluding protocols, reviews, commentaries, editorials, opinions, case studies and case reports, book chapters and dissertations. Publications in preprint were also excluded.
- *Timeframe*: Publications from 1 December 2019 to 18 August 2023. Studies needed to report specifically on reproducibility or generalizability of the approach, strategy, or intervention.

### Search strategy

We performed bibliographic database searches in CINAHL, Cochrane CENTRAL Register of Controlled Trials, EMBASE, ERIC, Education Research Complete, MEDLINE, and APA PsycINFO. Search strategies were developed in partnership with a librarian co-investigator (DLL) and a PRESS review was performed (ND) [21]. The full Medline search strategy is available in Supplemental Table 1. Manual de-duplication of records occurred in EndNote 20; remaining records were then uploaded and de-duplicated in Covidence.

### Record selection

Prior to title and abstract screening, researchers (CS, SJM, RBM, MC) reached an agreement > 90% in a calibration exercise including 50 random citations. Two researchers (CS; SJM, RBM, or MC) then independently screened all titles and abstracts in duplicate against the a priori defined eligibility criteria. Any study selected by a reviewer at this stage progressed to full text screening. Researchers (CS, SJM, RBM, MC) then completed a second calibration exercise (with 10 random full text articles) to reach an agreement > 90% before independently and in duplicate reviewing the full text of all records. Studies that were marked as eligible at this stage by both researchers were included in the review and proceeded to data charting. Discrepancies in record selection were resolved through discussion and inclusion of an external researcher (JPL) if necessary.

### Data charting

A comprehensive and relevant data charting table was developed and piloted by the study team (CS, SJM). The following data were abstracted independently and in duplicate by two researchers (CS, RBM): study characteristics (e.g., timeline, location, study design, number of sites), sample characteristics (e.g., sample size, age, gender), information on that strategy, approach, or intervention (e.g., empirical steps, required expertise, limitations), relevant outcomes (e.g., not mutually exclusive well-being framework domain and sub-domain(s)), pertinent recommendations (i.e., for future strategies, approaches, or interventions), and general conclusions as reported by the study authors. We contacted corresponding authors of included studies when further information or clarification was required.

In addition, we assessed strategies, approaches or interventions that were applied (i.e., *approach applied*) using a six-step model aimed at addressing our research objectives based on the method described by Kastner and colleagues [22]: (1) steps or guiding principles to conduct the approach (e.g., elements, or a step-wise protocol); (2) derivation of the approach from empirical evidence (i.e., if derived from observation and experiment, or published theory); (3) minimum expertise to conduct the approach (i.e., whether additional personnel are required [e.g., social worker, psychiatrist]); (4) limitations to the approach (e.g., dependence on specific materials, requirement of stable WiFi connection or personal device); (5) reproducibility of the approach (i.e., operationalized, evidenced by use in multiple sites); (6) feasibility of the approach to other contexts (i.e., generalizable, considering internal validity should precede external validity). Two reviewers (CS, RBM) independently in duplicate recorded notes for each of these six domains. Following discussion of discrepancies, a second round of summation by the same two reviewers (CS, RBM) was taken to reach 100% agreement.

### Data synthesis

Data charted from the included studies was synthesized quantitatively and qualitatively using the convergent integrated approach by the Joanna Briggs institute [19]. This approach refers to the process of combining charted data from quantitative studies and qualitative studies and involves data transformation into a mutually compatible format. For the current review, quantitative data was ‘qualitized’ such that data from quantitative studies was converted into textual descriptions to allow integration with qualitative data [23]; ‘qualitizing’ involved a narrative interpretation of the quantitative results into declarative stand-alone sentences to answer the review questions. We then assembled the ‘qualitized’ data with the qualitative data that was charted directly from included qualitative studies. Repeated and detailed examination of the assembled data occurred to identify categories on the basis of similarity in meaning within each domain and sub-domain of the underpinning framework. Where possible, categories were aggregated to produce overall integrated findings of the review. Data from included studies that presented new data on a well-being strategy, approach, or intervention (i.e., *approach applied*) was synthesized independent from studies that provided recommendations to inform development of a future well-being strategy, approach, or intervention (i.e., *approach suggested*).

## Results

**Fig. 1:**
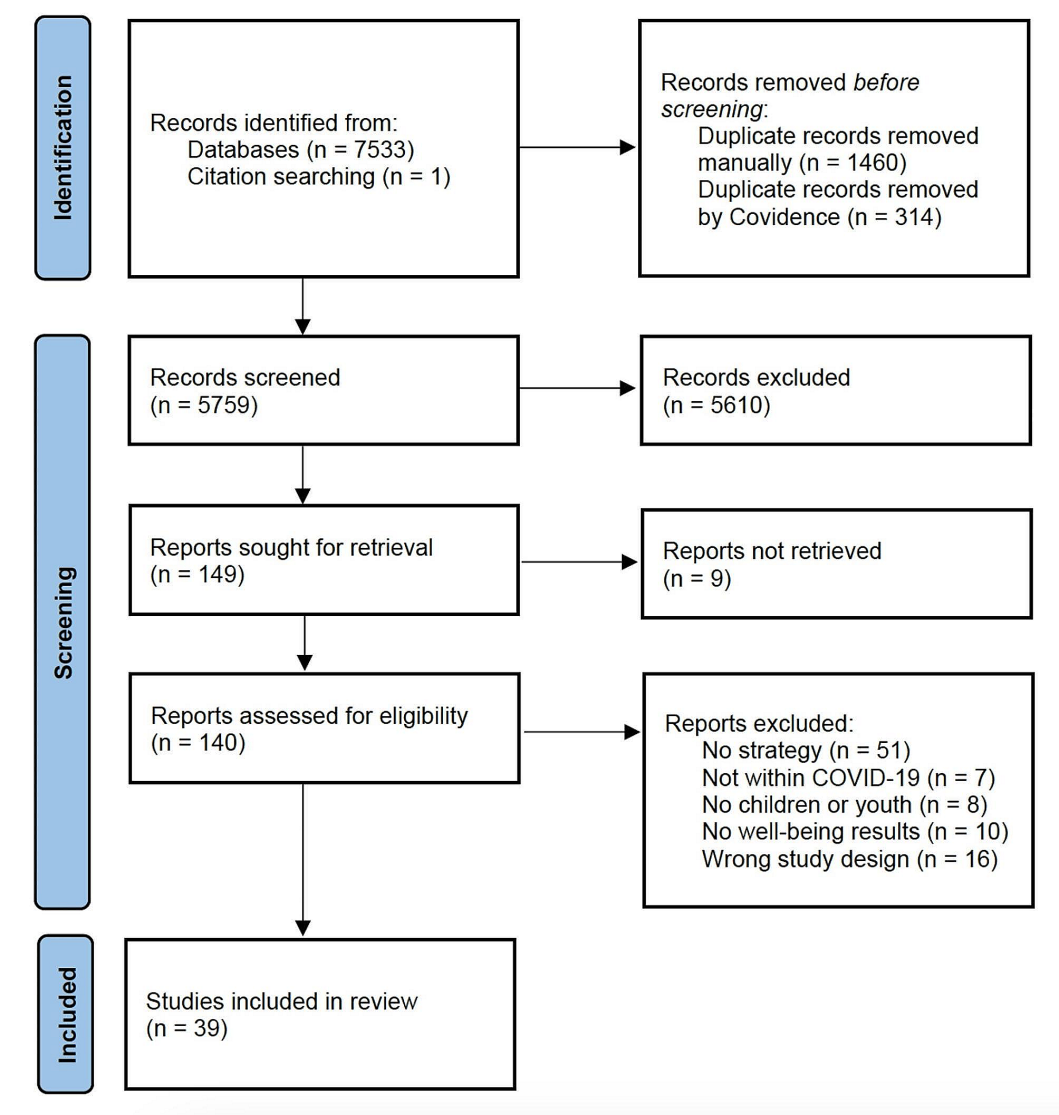
Record selection flow chart

We collected 7,533 records from searching databases and bibliographies of relevant papers (Fig. 1). After duplicates were manually deleted and automatically removed using Covidence, 5,759 unique records were left. Four researchers (CS, SJM, RB, and MC) excluded 5,610 records after screening titles and abstracts. 140 full text records were then assessed for eligibility and 101 studies were removed. Most studies were excluded at this stage because they did not assess or provide recommendations for a strategy, approach, or intervention (*n* = 51/101, 50.1%). The final number of records included in the scoping review was 39 [24–62]. Characteristics of included records organized by approach type (*applied* or *suggested*) then study design and first author are provided in Supplemental Table 2. In-depth descriptions of the approaches, strategies, or interventions to mitigate deleterious impacts on children and youth in future public health crises are provided in Supplemental Table 3 (*if categorized as approach applied*) or Supplemental Table 4 (*if categorized as approach suggested*).

### Description of included records

The majority of studies were categorized as *approach applied* (*n* = 22/39, 56.4%) compared to *approach suggested* (*n* = 17/39,43.6%) (Table 1). Most studies overall used a cross-sectional study design (*n* = 15/39, 38.5%), followed by experimental (*n* = 11/39, 28.2%) (including randomized controlled trials [*n* = 4/39, 10.3%], cluster randomized trials [*n* = 3/39, 7.7%] and quasi-experimental methodologies [*n* = 4/39, 10.3%]); *approach applied* records most frequently adopted an experimental design (*n* = 11/22, 50.0%), whereas *approach suggested* records most frequently adopted a cross-sectional design (*n* = 13/22, 59.1%). Twenty-one approaches (53.4%; *n* = 10/21, 47.6% *approach applied*) were self-guided while 17 approaches (41.0%; *n* = 12/17 *approach applied*, 70.6%) were guided synchronous by a trained professional; two approaches (5%, *n* = 2/2, 100% *approach suggested*) were unclear. Youth aged 13–18 years was the population studied most frequently overall (*n* = 27/39, 69.2%); *approach applied* records most frequently studied children (*n* = 9/17, 53.0%), whereas *approach suggested* records most frequently studied youth (*n* = 19/22, 86.4%). Overall, 7 studies (17.9%; 7/7, 100% *approach applied*) engaged children and youth in the development and refinement of approaches. Records commonly originated from the United States (*n* = 10/39, 25.6%), China (*n* = 5/30, 16.7%), or Canada (*n* = 4/39, 10.3%) (Supplemental Fig. 1); the majority of participants in 23 studies (58.9%) were White/Caucasian. The majority of included studies (*n* = 27/39, 69.2%) were conducted in 2021, with fewer studies conducted in 2022 and 2023 (*n* = 9/39, 23.1%; *n* = 3/39, 7.7%, respectively).

**Table 1:** Frequency of well-being domains targeted in the included records by age group and approach

Well-being domains^1^	Children (1–12 years)^2^ (*n* = 12)	Youth (13–18 years)^3^ (*n* = 27)	Approach applied^4^ (*n* = 17)	Approach suggested^5^ (*n* = 22)
**1) Good health and optimum nutrition**	**8 (66.7%)** ^6^	**19 (70.4%)**	**12 (70.6%)**	**15 (68.2%)**
Physical health and capacities	4 (50.0%)^7^	6 (31.6%)	5 (41.7%)	5 (33.3%)
Mental health and capacities	6 (75.0%)	18 (94.7%)	10 (83.3%)	14 (93.3%)
Optimal nutritional status and diet	0 (0.0%)	1 (5.3%)	0 (0.0%)	1 (6.7%)
**2) Connectedness**	**3 (25.0%)**	**18 (66.7%)**	**7 (41.2%)**	**14 (63.6%)**
Connectedness	3 (100.0%)	15 (83.3%)	5 (71.4%)	13 (92.9%)
Valued and respected by others and accepted as part of the community	1 (33.3%)	4 (22.2%)	1 (14.3%)	4 (28.6)
Attitudes	1 (33.3%)	1 (5.6%)	2 (28.6%)	0 (0.0%)
Interpersonal skills	1 (33.3%)	3 (16.7%)	4 (57.1%)	0 (0.0%)
Activity	0 (0.0%)	1 (5.6%)	1 (14.3%)	0 (0.0%)
Change and development	0 (0.0%)	2 (11.1%)	2 (28.6%)	0 (0.0%)
**3) Safety and a supportive environment**	**2 (16.7%)**	**9 (33.3%)**	**2 (11.8%)**	**9 (40.9%)**
Safety	1 (50.0%)	5 (55.6%)	1 (50.0%)	5 (55.6%)
Material conditions in the physical environment are met	1 (50.0%)	2 (22.2%)	0 (0.0%)	3 (33.3%)
Equity	1 (50.0%)	3 (33.3%)	1 (50.0%)	3 (33.3%)
Equality	1 (50.0%)	3 (33.3%)	1 (50.0%)	3 (33.3%)
Non-discrimination	1 (50.0%)	3 (33.3%)	2 (100.0%)	2 (22.2%)
Privacy	0 (0.0%)	0 (0.0%)	0 (0.0%)	0 (0.0%)
Responsive	0 (0.0%)	2 (22.2%)	1 (50.0%)	1 (11.1%)
**4) Learning, competence, education, skills, and employability**	**5 (41.7%)**	**13 (48.1%)**	**10 (58.8%)**	**8 (36.4%)**
Learning	1 (20.0%)	6 (46.2%)	1 (10.0%)	6 (75.0%)
Education	1 (20.0%)	1 (7.7%)	1 (10.0%)	1 (12.5%)
Resources	4 (80.0%)	7 (53.8%)	6 (60.0%)	5 (62.5%)
Skills	1 (20.0%)	5 (38.5%)	4 (40.0%)	2 (25.0%)
Employability	0 (0.0%)	0 (0.0%)	0 (0.0%)	0 (0.0%)
Confidence that they can do things well	0 (0.0%)	1 (7.7%)	1 (10.0%)	0 (0.0%)
**5) Agency and Resilience**	**4 (33.3%)**	**12 (44.4%)**	**8 (47.1%)**	**8 (36.4%)**
Agency	1 (25.0%)	8 (66.7%)	5 (62.5%)	4 (50.0%)
Identity	1 (25.0%)	5 (41.7%)	2 (25.0%)	4 (50.0%)
Purpose	1 (25.0%)	5 (41.7%)	4 (50.0%)	2 (25.0%)
Resilience	2 (50.0%)	9 (75.0%)	5 (62.5%)	6 (75.0%)
Fulfillment	2 (50.0%)	3 (25.0%)	2 (25.0%)	3 (37.5%)

^1^As described by Ross DA, Hinton R, Melles-Brewer M, et al. Adolescent well-being: a definition and conceptual framework. J Adolesc Health. 2020;67(4):472–476

^2^Includes ages that fall under 1–12 years of age. If age range included older ages, means younger than 12 were defined as children. If records did not report mean, median and IQR was used

^3^Includes ages that fall under 13–18 years of age. If age range included younger ages, means older and equal to 12 were defined as youth. If records did not report mean, median and IQR was used

^4^Approach suggested includes records that provided findings and/or recommendations that have the potential to inform a future approach to mitigate the deleterious impacts of the pandemic on children and youth well-being outcomes

^5^Approach applied includes records that implemented and reported on an approach to mitigate the deleterious impacts of the pandemic on children and youth well-being outcomes

^6^ Percentages demonstrate the total number of records that measured a well-being outcome divided by the total number of records. This is not a mutually exclusive value, for example records could have measured outcomes that fall under connectedness and agency and resilience

^7^ Percentages demonstrate the total number of records that measured a well-being subdomain divided by the total number of records related to their respective well-being domain. For example, the number of records related to the subdomain attitudes was divided by the total number of records related to connectedness. This is not a mutually exclusive value; record could have measured outcomes that fall under connectedness and attitudes

### Outcomes

Table 1 depicts included records by population, approach type (*applied* or *suggested*), and reported outcomes by framework domains and sub-domains. The most frequently reported outcomes were related to good health and optimum nutrition (*n* = 28/39, 71.8%), followed by connectedness (*n* = 22/39, 56.4%), learning, competence, education, skills, and employability (*n* = 18/39, 46.1%), agency and resilience (*n* = 16/39, 41.0%), and safety and a supportive environment (*n* = 11/39, 28.2%).

### Good health and optimum nutrition

#### Approach applied

The majority of all included studies assessed outcomes related to good health and optimum nutrition (*n* = 27/39, 69.2%). Of the studies that reported an *approach applied*, 70.6% (*n* = 12/17) reported an outcome of good health and nutrition. The most frequently reported subdomain assessed was mental health and capacities (*n* = 10/12, 83.3%), whereas no study assessed optimal nutritional status and diet (*n* = 0/17, 0.0%). Of the 12 studies with an *applied approach*, 75% (*n* = 9/12) described the steps of guiding principles, 91.7% (*n* = 11/12) were derived from empirical evidence, 83.3% (10/12) considered expertise, and 58.3% (*n* = 7/12, 58.3%) reported limitations, feasibility, and reproducibility (Table 2). Approaches most frequently aimed to improve cognitive behavioural, socio-emotional, resilient and adaptive skills and knowledge (i.e., mindfulness, positive education, time management) (*n* = 6/12, 50.0%). Mental and physical health outcomes that were most commonly reported as improved among the included studies were anxiety (*n* = 3/12, 25.0%), and physical activity or sedentary behaviour (*n* = 3/12, 25.0%) (Table 3).

**Table 2:** Summary of evidence on approaches, interventions, or strategies applied to mitigate deleterious impacts on children and youth during the COVID-19 pandemic by well-being domain

Questions used to guide evidence synthesis	Good Health and Optimum Nutrition^7^		Connectedness		Safety and a Supportive Environment		Learning, competence, education, skills, and employability		Agency and Resilience	
	*N* = 12		*N* = 7		*N* = 2		*N* = 10		*N* = 8	
Steps or guiding principles described^1^	9	75.0%	6	85.7%	2	100.0%	6	60.0%	4	50.0%
Derived from empirical evidence in full or part^2^	11	91.7%	5	71.4%	2	100.0%	8	80.0%	7	87.5%
Minimum expertise considered^3^	10	83.3%	4	57.1%	2	100.0%	6	60.0%	5	62.5%
Limitations reported^4^	7	58.3%	6	85.7%	2	100.0%	6	60.0%	3	37.5%
Approach is reproducible^5^	7	58.3%	5	71.4%	2	100.0%	6	60.0%	2	25.0%
Approach can be feasibly applied to other contexts^6^	7	58.3%	3	42.7%	2	100.0%	4	40.0%	1	12.5%

^1^Steps or guiding principles to conduct the approach

^2^Approach is derived from empirical evidence (i.e., through observation or experiment) or from published theory

^3^Minimum expertise required to conduct the approach

^4^Limitations to the approach

^5^Is the approach reproducible? (i.e., evidenced by use at multiple settings)

^6^Can the approach can be feasibly applied to other contexts?

^7^As described by Ross DA, Hinton R, Melles-Brewer M, et al. Adolescent well-being: a definition and conceptual framework. J Adolesc Health. 2020;67(4):472–476

**Table 3:** Common themes of the recommendations and findings presented in the included records

Themes^1^	Number of studies	List of studies by first author
**Good health and optimum nutrition** ^2^	**28**	
*Approach applied* ^*3*^	12 (42.9%)^5^	
Participation in artistic, community or sport-related activities	7 (58.3%)^6^	Caldwell, Han, Lemes, Lemos, Malbeouf-Hurtbuise Thompson
Aimed to improve cognitive behavioural, socio-emotional, resilient, and adaptive skills and knowledge	6 (33.3%)	Duan, Kishida, Lemos, Marques, Waters, Yuan
Improved mental health outcomes	4 (33.3%)	Duan, Kishida, Malboeuf-Hurtubise, Zheng
Improved anxiety	3 (25.0%)	Duan, Kishida, Zheng
Improved physical activity outcomes	3 (25.0%)	Caldwell, Han, Lemes
*Approach suggested* ^*4*^	16 (57.1%)^7^	
Physical exercise and school sports	6 (37.5%)^8^	Bourion Bédès, Cortes-Garcia, Grimes, LaForge-MacKenzie, McGuine, Zhu (2021 A)
Family and parental relationships	4 (25.0%)	Bourion Bédès, Silk, Wang, Zhu (2022)
School and other institutional support	3 (18.8%)	McCluskey, Parker, Zhu (2022)
Social media and technology	3 (18.8%)	Cortes-Garcia, Liang, Zhu (2021B)
Social connection and peer support	3 (12.5%)	Bourion Bédès, Cortes-Garcia, Jones
Nutrition	2 (18.8%)	Cortes-Garcia, Grimes
**Connectedness**	**22**	
*Approach applied*	7 (31.8%)	
Aimed to improve cognitive behavioural, socio-emotional, resilient, and adaptive skills and knowledge	4 (57.1%)	Kishida, Li, Marques, Özedemir, Paiva
Community support and civic involvement by youth	3 (42.9%)	Levestek, Paiva, Thompson
Improve socio-emotional skills and social support	3 (42.9%)	Kishida, Levestek, Li, Özedemir
Improve mental health outcomes	2 (28.6%)	Kishida, Levestek
Social connectivity through media	1 (14.3%)	Li
*Approach suggested*	15 (68.2%)	
School belonging, teacher support, and school connectedness	8 (46.7%)	Bryce, Gadermann, Arslan, Jones, Oinas, Parker, Soon, Zhu (2022)
Parenting and family relationships	7 (53.3%)	Gadermann, Arslan, Jones, Parker, Silk, Wang, Zhu (2021B)
Positive impact of social support	5 (13.3%)	Bourion Bédès, Bryce, Parker, Soon, Zhu (2021 A)
Social media, gaming, and technology	2 (33.3%)	Liang, Zhu (2021B)
**Safety and a supportive environment**	**11**	
*Approach applied*	2 (18.2%)	
Civic involvement by youth	2 (100.0%)	Paiva, Thompson
*Approach suggested*	9 (81.8%)	
Reporting science media safely and reliably	3 (33.3%)	Cortes-Garcia, Gadermann, Silk
Positive and accessible learning environment	3 (22.2%)	Oinas, Silk, Zhu (2022)
Empower students	2 (33.3%)	Godawa, Silk
Lack of support from schools	1 (11.1%)	McCluskey
**Learning, competence, education, skills, and employability**	**18**	
*Approach applied*	9 (50.0%)	
Aimed to improve cognitive behavioural, socio-emotional, resilient, and adaptive skills and knowledge	8 (88.8%)	Caldwell, Kishida, Lemos, Li, Özedemir, Thompson, Waters, Yuan
Techniques taught to cope with the pandemic	2 (22.2%)	Li, Marques
*Approach suggested*	9 (50.0%)	
Recommendation to teach cognitive behavioural, socio-emotional, resilient, and adaptive skills and knowledge	4 (44.4%)	Arslan, Cortes-Garcia, Oinas, Sciacca,
Strategies for teachers and staff	4 (44.4%)	Cortes-Garcia, McCluskey, Oinas, Silk
Recommendation to incorporate peer learning	3 (33.3%)	Cortes-Garcia, McCluskey, Oinas
Academic motivation assessed as a measure	2 (22.2%)	Bryce, Arslan
Importance of parenting on well-being	2 (22.2%)	Arslan, Sciacca
**Agency and resilience**	**16**	
*Approach applied*	8 (50.0%)	
Aimed to improve cognitive behavioural, socio-emotional, resilient, and adaptive skills and knowledge	8 (100.0%)	Gadari, Lemos, Levestek, Özedemir, Paiva, Waters, Yuan, Thompson
Increased resilience and adaptive skills	5 (12.5%)	Gadari, Levestek, Özedemir, Waters, Yuan
Increased civic engagement	2 (31.3%)	Paiva, Thompson
*Approach suggested*	8 (50.0%)	
Promote coping and resilience skills	6 (75.0%)	Bryce, Godawa, Ho, Soon, Wang, Zhu (2022)
Social support, connection, relationships, and resilience	6 (75.0%)	Gadermann, Godawa, Parker, Soon, Wang, Zhu (2022)
Establish agency and empowerment for student	3 (37.5%)	Godawa, Parker, Zhu (2022)
Hope and optimism as a protective measure	2 (25.0%)	Bryce, Gadermann
Resilience as a measure	2 (25.0%)	Ho, Zhu

^1^Themes among main findings, conclusions, and recommendations as identified by the research team

^2^Well-being outcomes as described by Ross DA, Hinton R, Melles-Brewer M, et al. Adolescent well-being: a definition and conceptual framework. J Adolesc Health. 2020;67(4):472–476

^3^Approach applied includes record that implemented and reported on an approach to mitigate the deleterious impacts of the pandemic on children and youth well-being outcomes

^4^Approach suggested includes record that provided findings and/or recommendations that have the potential to inform a future approach to mitigate the deleterious impacts of the pandemic on children and youth well-being outcomes

^5^Percentages demonstrate the total number of records that applied an approach divided by the total number of records categorized under their respective well-being domain

^6^Percentages demonstrate the total number of records that presented a theme divided by the total number of records under their respective well-being domain that applied an approach

^7^Percentages demonstrate the total number of records that applied an approach divided by the total number of records categorized under their respective well-being domain

^8^Percentages demonstrate the total number of records that presented a theme divided by the total number of records under their respective well-being domain that applied an approach

#### Approach suggested

Of the studies categorized as *approach suggested*, the majority (*n* = 15/22, 68.2%) assessed outcomes related to good health and optimum nutrition. The most common sub-domain reported among these studies was mental health and capacities (*n* = 14/15, 93.3%), followed by physical health and capacities (*n* = 5/15, 33.3%), and optimal nutritional status and diet (*n* = 1/15, 6.7%). The most common recommendations among studies that assessed mental health outcomes were improving family relationships, parental-wellbeing, and home environments (*n* = 4/22, 18.1%). This was closely followed by recommendations to: facilitate in-school and other institutional mental health supports (e.g., community, religious institutions) (*n* = 3/22, 13.6%), support social connections among peers and trusted adults to build a sense of belongingness (*n* = 3/22, 13.6%) and to moderate children and youth’s use of technology and media (*n* = 3/22, 13.6%), such as their exposure to science media (*n* = 2/22, 9.1%) or regulating gaming practices (*n* = 1/22, 4.5%). Studies largely recommended increasing physical activity and decreasing sedentary behaviour in children and youth (*n* = 6/22, 27.3%) for enhanced physical health outcomes. Other common recommendations for improving physical health were to continue facilitating school sports (*n* = 2/22, 9.1%) and providing online or at-home exercise programs despite the eventual pandemic end (*n* = 3/22, 13.6%). Two studies that *suggested* an *approach* focused on nutritional health outcomes (*n* = 2/22, 9.1%); authors recommend developing nutritional interventions that encourage balanced diets (*n* = 1/22, 4.5%), but are still fun and engaging, such as cooking challenges or using social media to engage students (*n* = 1/22, 4.5%).

### Connectedness

#### Approach applied

Over half of all included studies reported outcomes related to connectedness (*n* = 21/39, 53.9%). Among studies categorized as *approach applied* (*n* = 7/17, 41.2%), the most common subdomains assessed were connectedness (*n* = 5/7, 71.4%) and interpersonal skills (*n* = 4/7, 57.1%). Further, 85.7% (*n* = 6/7) of *approach applied* studies described the steps of guiding principles and limitations, 71.4% (*n* = 5/7) were derived from empirical evidence and were reproducible, 57.1% (*n* = 4/7) considered expertise, and 42.9% (*n* = 3/7) reported on feasibility. A frequently reported goal among the *approach applied* was to improve socio-emotional, resilient, and adaptive skills and knowledge (*n* = 4/7, 57.1%); all four of these studies found improvements in at least one skill measured. Two studies reported on increased community support from peers and teachers and civic involvement from youth (*n* = 2/7, 28.6%). One record identified the impact of social media and technology on connectedness as a method for improved social connectivity; however, this depended on the purpose and type of use (*n* = 1/7, 14.3%).

#### Approach suggested

Of the *approach suggested* studies, 63.6% (*n* = 14/22) measured an outcome related to connectedness. The most common sub-domain reported was connectedness (*n* = 13/14, 92.9%), followed by feeling valued and respected by others and accepted as part of the community (*n* = 4/14, 28.6%). Among the *approach suggested* studies, school belonging and connectedness including support from and connections with teachers was most frequently described as a potentially important factor for improved child and youth health during the pandemic (*n* = 8/14, 57.1%). Better social support for children and youth was recommended by authors because it was found to have managed stress and improved quality of life during the pandemic (*n* = 5/14, 35.7%). Notably, one study found that half of respondents reported no changes and reported better social support during the pandemic. During the pandemic, parental and family support was suggested to be important for child and youth wellbeing (*n* = 7/14, 50.0%), especially emotional health outcomes (*n* = 5/7, 71.4%). Thus, it was recommended by authors to remind parents of their role in their children’s emotional health (*n* = 1/7, 14.3%), to spend time with them (*n* = 1/7, 14.3%), and to facilitate open conversations (*n* = 2/7, 28.6%). In one study, parental supervision for gaming during limited leisure options was recommended (*n* = 1/14, 7.1%), whereas another study identified social media and technology as conduits for enhanced social connectivity (*n* = 1/14, 7.1%).

### Learning, competence, education, skills, and employability

#### Approach applied

Eighteen studies (*n =* 18/39, 46.2%) overall reported on outcomes related to learning, competence, education, skills, and employability (Table 1). Of the studies that *applied* an *approach* (*n* = 10/18, 55.6%), most aimed to improve child and youth resources, life skills, and competencies (*n* = 6/10, 60.0%) or skills (*n* = 4/10, 40.0%). These resources included social and emotional skills (*n* = 2/10, 20.0%), adaptive coping skills such as mindfulness (*n* = 2/10, 20.0%), skills to increase health and health equity literacy (*n* = 1/10, 10.0%), and positive education skills (*n* = 1/10, 10.0%). Creativity through music was used in one of the (10.0%) included studies. Among records that *applied* an *approach*, 60.0% were reproducible, described the steps of guiding principles and limitations, and considered the minimum expertise (*n* = 6/10), 80.0% were derived from empirical evidence (*n* = 8/10, 80.0%), and 40.0% reported feasibility (*n* = 4/10, 40.0%).

#### Approach suggested

An *approach* was *suggested* within eight included studies (*n* = 8/18, 44.4%). Common recommendations were to develop students’ self-regulation (1/8, 12.5%), socio-emotional (1/8, 12.5%), and adaptive skills and knowledge such as teaching children and youth to improve their online skills (1/8, 12.5%) and to use their strengths (*n* = 1/8, 12.5%). Studies highlighted the importance that educational staff, peers, and parents have in improving well-being (*n* = 6/8, 50.0%), specifically academic learning and motivation (*n* = 3/6, 50.0%). Two studies suggested that academic motivation may increase after strength-based parenting and may contribute to feelings of school belonging and hope (*n* = 2/8, 25.0%).

### Agency and resilience

#### Approach applied

Sixteen studies reported on outcomes related to agency and resilience (*n* = 16/39, 41.0%). Of these records that *applied* an *approach* (*n* = 8/16, 50.0%), the most common subdomains described were agency (*n* = 5/8, 62.5%), resilience (*n* = 5/8, 62.5%), and purpose (*n =* 4/8, 50.0%). All eight of these studies aimed to improve cognitive behavioural (*n* = 3/8, 37.5%), socio-emotional (*n* = 7/8, 87.5%), resilient (*n* = 4/8, 50.0%) or adaptative skills, or knowledge (*n* = 5/8, 62.5%). One study implemented an intervention specific to improving resilience by an online educational intervention teaching problem-solving and socio-emotional skill and found a significant increase in self-reported social efficacy (*n* = 1/8, 12.5%). Half of the studies that reported on outcomes related to agency and resilience also reported improved social and emotional skills (*n* = 4/8, 50.0%) including intra-personal outcomes (*n* = 1/8, 12.5%), emotional resilience (*n* = 2/8, 25.0%), and problem-solving (*n* = 1/8, 12.5%). Two studies described how meaningful youth involvement and health equity literacy added to youth agency and their desire to participate in social advocacy (*n* = 2/8, 25.0%). Half (50.0%) of the studies that *applied* an *approach* described the steps of guiding principles (*n* = 4/8), while 87.5% were derived from empirical evidence (*n* = 7/8), 62.5% considered expertise (*n* = 5/8), 37.5% reported limitations (*n* = 3/8), 25% were reproducible (*n* = 2/8), and 12.5% reported feasibility (*n* = 1/8).

#### Approach suggested

Eight records were categorized as an *approach suggested* (*n* = 8/16, 50.0%). Common subdomains described were resilience (*n* = 6/8, 75.0%), agency (*n* = 4/8, 50.0%), and identity (*n* = 4/8, 50.0%). Of these studies, two recommended prioritizing hope skills and optimism among children and youth as this could potentially increase academic motivation (*n* = 2/8, 25.0%). Another common recommendation was to encourage education on coping and resilience skills (e.g., positive thinking, acceptance) (*n* = 6/8, 75.0%) to empower children and youth to organize personal activities and to allow them to participate in social discussions; two studies (25.0%) recommended this to specifically mitigate stress and to avoid less healthy ways of coping (*n* = 2/8, 25.0%). One study suggested that increased resilience may be associated with lower academic stress and higher life satisfaction (*n* = 1/8, 12.5%), while another suggested that increased resilience may buffer the impact of mental health challenges in younger students (*n* = 1/8, 12.5%).

### Safety and a supportive environment

#### Approach applied

Safety and a supportive environment was the domain least represented among the included records (*n* = 11/39, 28.2%). The two studies that *applied* an *approach* aimed to improve safety and a supportive environment by involving students in civic engagement such as the dissemination of materials that would conceivably foster social responses and increase their health equity literacy (*n* = 2/11, 18.2%). Both studies described steps or guiding principles, were derived from empirical evidence, considered expertise, and reported feasibility, limitations, and reproducibility (*n* = 2/2, 100%, all).

#### Approach suggested

Nine studies that *suggested* an *approach* reported outcomes related to safety and a supportive environment study (*n* = 9/22, 40.9%) (Table 1). Many studies described the importance of a positive environment (*n* = 3/9, 33.3%), which included from peer learning opportunities (*n* = 1/9, 11.1%), quality schooling (*n* = 1/9, 11.1%), resources and space (*n* = 2/9, 22.2%), and accessible and equitable learning (*n* = 2/9, 2.2%). Negative environments, such as experiencing peer victimization and a lack of support from schools’ post-pandemic (*n* = 1/9, 11.1%), were found to be detrimental to students’ mental health during the pandemic (*n* = 2/9, 22.2%). Recommendations to mitigate these negative environments included mental health screening and checking in regularly with students (*n* = 1/9, 11.1%) and for more reflection and conversation post-pandemic allowing conversations related to mental health to become the new norm (*n* = 1/9, 11.1%). Another common recommendation to ensure safe environments for children and youth was the use of science media for safe and reliable media exposure (*n* = 3/9, 33.3%); two recommended leveraging students’ voices to provide them the agency to manage their own tasks to reduce school-related stress (*n* = 2/9, 22.2%).

## Discussion

We have synthesized the evidence to provide a detailed review of well-being strategies, approaches, and interventions targeted to improve child and youth health during the COVID-19 pandemic and into the post-COVID-19 pandemic period. Despite that the majority of studies (22/39) reported on approaches that were applied and tested, few studies engaged children and youth in the development and refinement of approaches; the rapid onset and unpredictability of the COVID-19 pandemic may have precluded meaningful engagement despite widespread recognition that early engagement can enhance usefulness and acceptability of innovations. Several studies reported on needs assessments—often in the form of cross-sectional surveys—to determine areas of focus for future approaches, strategies, and interventions. Most of these studies recommended complex approaches with various components that may challenge our ability to delineate efficacious parts. Additional research is required to assess necessary adaptations to existing well-being approaches, strategies, and interventions to support child and youth health as we enter the post-COVID-19 pandemic period and prepare for future public health crises.

Fostering social and community-based relationships is essential to nurture positive child and youth well-being. It has long been demonstrated that children and youth who report meaningful connections with their parents, teachers, and school environments have lower levels of mental health difficulties [63, 64]. The included studies highlighted that connectedness during the COVID-19 pandemic had a significantly positive impact on child and youth health [24, 65–67], and personal relations among children and youth with their educational teachers, parents, and peers was a hallmark of most included studies. Accordingly, dedicated time for social activity in educational settings that includes opportunities to connect with teachers and peers was one approach that was frequently referred to as necessary to continue post-COVID-19, which may occur through facilitating open discussions [66, 68], conducting team-based activities [66], or enhancing peer learning activities [69, 70]. Zhu et al., (2022), demonstrated that enhanced positive teacher and student relationships mitigated the impacts of peer victimization and mental health difficulties for younger students during the pandemic; these relationships were also defined by parental mediation and approaches [24, 71]. The pandemic revealed that students’ social-emotional development is inadequately supported even in normal times, calling for an urgent need for more effective social-emotional learning opportunities and innovative approaches to expand student supports. What is needed is an integrated and responsive system of education and tailored supports that can flexibly meet each individual student’s highly variable needs on an immense, post-pandemic scale. The data also indicates the complexities of connectivity through social media and technology. Although social media provides a channel for connection and it was found to provide relief from pandemic-related stressors, it may have in turn added to stress surrounding current events and may have increased opportunities for cyber bullying as well as screen time [43, 72]. Moving beyond COVID-19, parents and teachers are suggested to educate children and youth on digital health literacy and skills and to monitor screen time to ensure media is used as a tool and not a crutch [73]. It is essential that policymakers commit to reporting science media safely and soundly to children and youth. In a post-COVID-19 era, these lessons continue to apply to bolster children and youth well-being, especially as technology continues to evolve and grow.

Continuing to educate children and youth with age-appropriate information to empower them to be informed stewards of their own health in the post-COVID-19 pandemic period was a common recommendation among included articles. The most frequently reported, long-term approaches with regard to continuously improved health literacy were comprised of complementary components for holistic well-being interventions that aided in the future protection against mis- and dis-information. Such components included cognitive-behavioral practices [74–76], positive thinking [57, 74] and strength-based thinking [29], as well as emotional regulation [77], interpersonal [30, 57, 74, 75], organizational [77], mindfulness [58, 76], and problem-solving skills [30, 77, 78]. It was suggested that adapting coping strategies that were initiated and integrated during the COVID-19 pandemic to the post-COVID-19 pandemic period could facilitate efficient increases in health literacy [55, 76, 77]. Further research is needed to better understand how to effectively individualize well-being approaches that target health literacy to protect child and youth health from continued adverse impacts related to information and communication challenges that were experienced in COVID-19.

Work is also needed to involve children, youth, and their parents in a collaborative, co-design process to thoughtfully provide adequate resources and the necessary infrastructure to ensure high fidelity and feasibility of interventions. This will help policymakers and service providers access high-quality evidence for resource allocation, universal and targeted intervention approaches, and mitigation strategies for Canadians during future pandemics.

Our study has several strengths. First, we searched multiple databases using a search strategy that was developed in partnership with a health research librarian to ensure a rigorous and reliable search; our selection process was intentionally broad to ensure all potentially relevant records were captured. Second, we included records reporting on any study design that provided quantitative and/or qualitative original data. This breadth of included and complementary data allowed us to comprehensively report on the extant literature. Third, we grounded the methods of our review in a widely published framework from The Partnership for Maternal, Newborn & Child Health and the World Health Organization of the United Nations H6 + Technical Working Group on Adolescent Health and Well-Being consensus framework for defining, programming, and measuring adolescent wellbeing, which is also part of a broader program of work that includes a multistakeholder Call to Action to prioritize adolescent well-being. Fourth, we assessed strategies, approaches or interventions that were applied using a six-step model based on the method described by Kastner and colleagues [22]. Despite these strengths, our study presented limitations. First, most studies were conducted in the United States and thus may present an inadequate representation of global findings, as school-wide isolation policies and COVID-19 incidence varied across countries and regions. Second, our results have limited longitudinal applicability as the majority of studies were conducted in the initial (early) phases of the pandemic; we cannot generalize our findings to changes in health that occurred in the later- and post-pandemic periods, although research in this area is needed to continue. Third, few included studies targeted equity-deserving and/or marginalized community in underserved communities. It is critical that future research focuses on these priority populations to better understand and adapt to their pandemic experiences and perspectives. Finally, despite our expert-developed search strategy and extensive database search, we did not search the grey literature that may have resulted in potentially relevant articles (e.g., governmental and non-governmental organization reports) being missed.

## Conclusions

This scoping review identified varied published strategies, approaches, or interventions that were primarily targeted to improve connectedness, belonging, and socialization, while grounded in methods to educate, engage, and empower children and youth in learning resilience, developing social and emotional skills, and practicing adaptive coping strategies. Studies that described synchronous approaches most often reported improved well-being among children and youth largely in the domain of good health and optimum nutrition, specifically physical and mental health. Further research that includes meaningful engagement of diverse children and youth is required to understand preservation of the critical roles of families, parents, teachers, and other peers with regard to child and youth health in the post-COVID-19 pandemic period to reach sub-groups of children and youth who are most at risk of negative well-being outcomes.

### Electronic supplementary material

Below is the link to the electronic supplementary material.

Supplementary Material 1

Supplementary Material 2

## Data Availability

The datasets analyzed are available from the corresponding author on reasonable request.

## Abbreviations

APA: American Psychological Association
COVID-19: Coronavirus Disease 2019
ERIC: Education Resources Information Centre
PRESS: Peer Review of Electronic Search Strategies
WHO: World Health Organization
