# Supplementary material for: Well-being approaches targeted to improve child and youth health post-COVID-19 pandemic: a scoping review
Source: BMC Health Serv Res. 2024 Jun 21;24:758. doi: 10.1186/s12913-024-11140-7 (PMC11193255; doi:10.1186/s12913-024-11140-7)
Supplement: Supplementary file 1 — Supplementary Material 1 [file 12913_2024_11140_MOESM1_ESM.docx]

**Supplemental Table 1.** Search strategy used in Medline.

| **Search component** | **Search terms** |
| --- | --- |
| COVID-19 pandemic | 1 COVID-19/ or SARS-CoV-2/ 2 (coronavirus/ or betacoronavirus/ or coronavirus infections/) and (disease outbreaks/ or epidemics/ or pandemics/) 3 (nCoV* or 2019nCoV or 19nCoV or COVID19* or COVID or SARS-COV-2 or SARSCOV-2 or SARS-COV2 or SARSCOV2 or SARS coronavirus 2 or Severe Acute Respiratory Syndrome Coronavirus 2 or Severe Acute Respiratory Syndrome Corona Virus 2).tw,kf. 4 ((new or novel or "19" or "2019" or Wuhan or Hubei or China or Chinese) adj3 (coronavirus* or corona virus* or betacoronavirus* or CoV or HCoV)).tw,kf. 5 (long COVID* or longCOVID* or postCOVID* or post-COVID* or postcoronavirus* or post-coronavirus* or postSARS* or post-SARS*).tw,kf. 6 ((coronavirus* or corona virus* or betacoronavirus*) adj3 (pandemic* or epidemic* or outbreak* or crisis)).tw,kf. 7 ((Wuhan or Hubei) adj5 pneumonia).tw,kf. 8 1 or 2 or 3 or 4 or 5 or 6 or 7 9 limit 8 to yr="2019 -Current" |
| Children and youth | 10 animals/ not humans/ 11 9 not 10 12 adolescent/ or child/ 13 (adolescen* or child or children or teen* or youth*).tw,kf. 14 12 or 13 15 11 and 14 16 limit 11 to ("child (6 to 12 years)" or "adolescent (13 to 18 years)") 17 15 or 16 |
| School social distancing policies | 18 ((parks or play or playground*) adj5 (cleaning or closure* or closed or distancing or restrict* or limit* or masks or masking or shutdown* or shut down*)).tw,kf. 19 ((college* or school* or universit*) adj5 (cleaning or closed or closure* or distancing or interrupt* or lockdown* or limit* or masks or masking or restrict* or saniti* or shutdown* or shut down* or suspen* or ventilat* or (covid* adj2 test*) or (covid* adj2 vaccin*))).tw,kf. 20 (in-person learning adj5 (interrupt* or limit* or restrict* or suspen*)).tw,kf. 21 home schooling.tw,kf.  22 ((at home or hybrid or online or remote) adj (educat* or learning)).tw,kf. 23 ((extracurricular or extra-curricular or intramural or school* or weekend) adj5 (activities or clubs or hobbies or sports or team or athletes or groups)).tw,kf. 24 18 or 19 or 20 or 21 or 22 or 23 25 17 and 24 26 limit 25 to (comment or editorial or letter)  27 25 not 26 |

**Supplemental Figure 1**. Well-being domains represented among the included records by country.

**Supplemental Table 2.** Characteristics of included records organized by approach type then study design and first author.

| **Approach applied^1^ or approach suggested^2^** | **Study design** | **Country** | **Population^3^** | **Data collection period** | **Age of youth (Years)** | **First author** | **Year** |
| --- | --- | --- | --- | --- | --- | --- | --- |
| Approach applied | Cross-sectional survey | Portugal | Children | June-20 to Jul-20 | M = 11.1 (SD = 2.2) Age Range: 8 to 15 | Lemos, G. C. | 2022 |
| Approach applied | Cross-sectional survey | Australia | Youth | Not reported | M = 14.8 (SD = 1.6) Age Range: 11 to 18 | Waters, L. | 2021 |
| Approach applied | Experimental - Cluster randomized trial | United States | Children | 19-Dec | Not reported | Li, L. | 2021 |
| Approach applied | Experimental - Cluster randomized trial | Canada | Children | May-20 to Jun-20 | M = 11.3 (SD = not reported) Age Range: not reported | Malboeuf‑Hurtubise, C. | 2021 |
| Approach applied | Experimental - Cluster randomized trial | China | Youth | 20-Mar | M = 13.5 (SD = 0.5)  Age Range: 12 to 13 | Zheng, Y. | 2021 |
| Approach applied | Experimental - Quasi-experimental | China | Children | May-20 to July-20 | M = 10.7 (SD=0.5) Age Range: 10 to 12 | Duan, W. | 2022 |
| Approach applied | Experimental - Quasi-experimental | China | Children | Oct-20 to Dec-20 | M Experimental Group: 4:2 ± 0:6 M Control Group: 4:3 ± 0:4 Age range not reported | Han, X. | 2022 |
| Approach applied | Experimental - Quasi-experimental | Japan | Youth | Jun-20 to Jul-20 | M =12.3 (SD=0.5) Age range not reported | Kishida, K. | 2022 |
| Approach applied | Experimental - Quasi-experimental | United States | Youth | Feb-21 to May-21 | High school students Mean and age range not reported | Thompson, H. R | 2022 |
| Approach applied | Experimental - Randomized control trial | Iran | Children | June-20 to Sept-20 | Mean not reported Age Range: 9 to 10 years | Gadari, S. | 2022 |
| Approach applied | Experimental - Randomized control trial | Brazil | Children | May-20 and Dec-2020 | M Boys: 9.4 (±1.8)  M Girls: 9.4 (±1.7)  Age range not reported | Lemes, V. B. | 2022 |
| Approach applied | Experimental - Randomized control trial | Northern Cyprus | Children | Unknown | Fourth and fifth graders Mean and age range not reported | Özedemir, M. B. | 2022 |
| Approach applied | Experimental - Randomized control trial | China | Youth | Feb-20 to Aug-20 | M Experimental: 13.3 (SD = 0.8) M Control: 13.5 (SD = 0.8) Age Range: 12 to 14 | Yuan, Y. | 2021 |
| Approach applied | Mixed methods | Canada | Children | Dec-20 to Feb-21 | M5 = 9.3 (SD6 = not reported) Age range not reported | Caldwell, H. | 2022 |
| Approach applied | Mixed methods | United Kingdom | Youth | Not reported | Mean not reported Age Range 8-20 | Levstek, M. | 2021 |
| Approach applied | Mixed methods | United Kingdom | Youth | Apr-20 to Jun-20 | M = 17.0 (SD = not reported) Age Range: 16 to 18 | Marques, S. S. | 2021 |
| Approach applied | Qualitative^4^ | Brazil | Youth | Oct-19. | Senior high school students Mean and age range not reported | Paiva, V. | 2021 |
| Approach suggested | Cross-sectional survey | Australia | Youth | 2022 | M = 14.8 (SD = 1.59) Age Range: 11 to 18 | Arslan, G. | 2022 |
| Approach suggested | Cross-sectional survey | France | Youth | May-20 to Jul-20 | M = 12.9 (SD = 3.0) Age range not reported | Bourion‑Bédès, S. | 2022 |
| Approach suggested | Cross-sectional survey | Canada | Children | Jan-20 to Mar-20 and Jan-21 to Mar-21 | M Time 1: 13 (SD = 0.1) M Time 2: 14 (SD = 0.1) Age range not reported | Gadermann A. | 2022 |
| Approach suggested | Cross-sectional survey | Poland | Youth | 20-Apr | M = 17 (SD= not reported) Age Range: 14 to 20 | Godawa, G. | 2021 |
| Approach suggested | Cross-sectional survey | Vietnam | Youth | Not reported | M = 15.4 (SD = 1.7) Age Range: 13 to 18 | Ho T. | 2022 |
| Approach suggested | Cross-sectional survey | United States | Youth | Jan-21 to Jun-21 | Not reported | Jones S.E. | 2022 |
| Approach suggested | Cross-sectional survey | Canada | Children | Nov-20 and Feb-21 to Mar-21 | M = 10.8 (SD = 3.8) Age range not reported | LaForge-MacKenzie, K. | 2022 |
| Approach suggested | Cross-sectional survey | United States | Youth | 20-Oct | M = 15 ± 1.2  Age range: 13–19 | McGuine, T. | 2022 |
| Approach suggested | Cross-sectional survey | Finland | Youth | 20-May | Mean not reported Age Range: 13 to 16 | Oinas, S. | 2022 |
| Approach suggested | Cross-sectional survey | Ireland | Youth | Jul-20 and Aug-20 | M = 13.8 (SD = 2.0)  Age Range: 10 to 17 | Sciacca, B. | 2022 |
| Approach suggested | Cross-sectional survey | China | Youth | 2022 | Fourth to eleventh graders Mean and age range not reported | Zhu, Q. | 2022 |
| Approach suggested | Cross-sectional survey | Hong Kong | Youth | 20-Jul | M = 12.6 (SD = 1.3) Age Range: 9-17 | Zhu, S. | 2021 |
| Approach suggested | Cross-sectional survey | Hong Kong | Youth | 20-Jun | M = 12.6 (SD = 1.3) Age Range: from 8 to 17 | Zhu, S. | 2021 |
| Approach suggested | Mixed methods | United States | Youth | 21-Feb | M = 14.5 (SD = 1.9) Age range not reported | Bryce, C. I. | 2022 |
| Approach suggested | Prospective cohort | United States | Youth | Apr-20 to May-20 | M = 15.1 (SD = 1.2) Age Range: 12 to 17 | Silk, J. S. | 2022 |
| Approach suggested | Prospective cohort | United States | Youth | 21-Apr | M = 15.0 (SD = not reported) Age Range: 13 to 18 | Wang, M. | 2021 |
| Approach suggested | Qualitative | United States | Youth | Dec-19 and Jun-20 to Aug-20 | Median = 13  Age Range: 11 to 15 | Grimes A. | 2022 |
| Approach suggested | Qualitative | United States | Children | 20-May | Mean not reported Age Range: 10 to 14 | Cortes-Garcia, L. | 2021 |
| Approach suggested | Qualitative | United States | Youth | Sept-20 to Dec-20 | M = 15.9 Age Range: 13 to 17 | Liang, E. | 2022 |
| Approach suggested | Qualitative | Scotland | Youth | Aug-20 to Sept-20 | Mean not reported Age Range: 14 to 18 | McCluskey, G. | 2021 |
| Approach suggested | Qualitative | United States | Youth | Jun-20 to Jul-20 | M = 15.2 (SD = not reported)  Age range: 12 to 17 | Parker, J. S. | 2021 |
| Approach suggested | Qualitative | Singapore | Youth | Oct-20 to Mar-21 | 14 to 15 (78%), 15 and above (22%) Age Range: 14 to 15 | Soon, T. C. | 2023 |

^1^Approach applied includes records that implemented and reported on an approach to mitigate the deleterious impacts of the pandemic on children and youth well-being outcomes.

^2^Approach suggested includes records that provided findings and/or recommendations that have the potential to inform a future approach to mitigate the deleterious impacts of the pandemic on children and youth well-being outcomes.

^3^Children are defined by participants 1-12 years of age. Youth are defined by participants 13-18 years of age.If age range included older ages, means younger than 12 were defined as children. If studies did not report mean, median and IQR was used.

^4^Qualitative records described research methodologies ethnography, grounded theory, phenomenology, interpretive description, and data collection methods such as focus groups, interviews, etc.

^5^Mean

^6^Standard deviation

**Supplemental Table 3.** Description of interventions to mitigate the deleterious impacts of the COVID-19 pandemic on children and youth.

| **First Author** | **Population^1^** | **Name** | **Description^2^** | **Steps^3^** | **Derivation^4^** | **Expertise^5^** | **Limitations^6^** | **Reproducible^7^** | **Feasible^8^** | **Author’s Conclusions and Recommendations^9^** |  |
| --- | --- | --- | --- | --- | --- | --- | --- | --- | --- | --- | --- |
|  |  |  |  |  |  |  |  |  |  |  |  |
|  |  |  |  |  |  |  |  |  |  |  |  |
| Caldwell, H. | Children | Build Our Kids' Success (BOKS) | BOKS consists of a variety of free, fun, and engaging resources to support children to develop a lifelong commitment to health and wellness. | Program leaders delivered full lesson plans, short movement bursts and movement-based games, activities, and resources on average for 70 minutes per week. | Theory | Educational leadership, training provided | Program leaders required training | Yes | Yes | BOKS suggested to be a protective factor of physical inactivity during the pandemic for children and successfully improved physical activity. For many, BOKS was their only source of physical activity. Although this program was easy to implement, future recommendations to consider were improving inclusivity (more challenging for older children, and simpler for younger children) and to emphasize children and youth's enjoyment when developing activities. |  |
| Duan, W. | Children | Strength-informed Acceptance and Commitment Therapy (SACT) | This online SACT intervention targets participants adaptability, to stay in the present moment, and practices healthy behaviours that promote a more positive life using a strengths perspective. SACT focuses on individual potential and strengths by learning to use one's strengths abilities to face adversity. | The intervention was organized as 10 lessons (45 minutes) held once a week for 10 weeks. The intervention consisted of five activities:  1. Activity 1 - Identifying Personal Strengths (1 lesson): By watching videos and thinking about the strengths of the characters, participants’ self-awareness of their personal strengths was promoted, and their cognition of their strengths was further reconstructed. 2. Activity 2 - Best Debater (2 lessons): conducting a debate on the pros and cons of online learning at home for students during the COVID-19 pandemic and identifying facts, ideas, and opinions, the students were required to use facts to support their opinions 3. Activity 3 - Gratitude Diary (2 lessons): writing down the three good things that happened every day and recording their feelings 4. Activity 4 - Pursuing the Beauty of Ideals (2 lessons): recording three persons or things they found beautiful and their experiences every week 5. Activity 5 - Plan and Implement (2 lessons): accomplishing something the students once wanted to do but did not do or something they currently wish to pursue 6. Concluding lesson - 1 lesson | Empirical and theory | Developed by social workers, educators, and psychologist. Program leader expertise were unspecified. | Technology equipment needed, privacy protection risk | Yes | Yes | SACT participants who have experienced crisis events experienced a significant reduction in anxiety symptoms and improvement of quality-of-life levels three months after the intervention, suggesting SACT is beneficial even long term. Caution is required when interpreting these results due to the small sample size. |  |
| Gadari, S. | Children | Resilience training | The virtual resilience training aimed to improve participants social self-efficacy including social ability, problem-solving and critical thinking skills, to prevent social and psychological harm in children. | 1. Questionnaires were sent into two groups. 2. The training was conducted in the forms of video and audio clips, videos, animations, child scenarios, text messages, storytelling, and questions and answers. Sometimes animations were sent before educational clip to brainstorm and find out students’ strengths and weaknesses, and they were asked to comment. Storytelling was used in some sessions for more impact and understanding of the subject. The researcher sometimes asked students to do some homework and then settled their problems.  Resilience training for 6 weeks, twice a week = 12 sessions (1) Orientation, (2) Self-awareness, (3) Problem-solving skills, (4) Problem-solving skills, (5) Responsibility, (6) Identify positive and negative thoughts, (7) Communication, (8) Friendship and making friends, (9) Positive and negative emotions, (10) Relaxation exercises and how to cope with stress, (11) Empathy, helping friends, and family, (12) Review and post-test. | Theory | Social medicine specialist, researchers | Technology equipment needed (computer and internet) | Not reported | Not reported | Findings showed a significant increase in social efficacy levels in girls in the intervention group immediately and one month after intervention. In comparison, there were no significant differences in the control group. Since this study focused on girls, future research should understand SACT's influence on boys. |  |
| Han, X. | Children | Family-based physical activity intervention | The 8-week app-based physical activity intervention used WeChat to share short videos to guide goal-directed family parent-child cooperative exercise for 30 minutes. Physical activity exercises included from running, climbing, jumping, throwing, flexibility, and coordination. | 1. Groups received 30 min of family PA intervention twice a week (= 8 hours of PA total) executed through an established official account “YOUXUE UP” loaded on the WeChat app. 2. The researchers established a class through the WeChat official account “YOUXUE UP” and sent a class invitation link to the parents in the EG. After the parents registered successfully, they entered the class group.  3. During the experimental intervention, the researchers sent preprepared practice tasks (short videos) to parents on time every Tuesday and Saturday (16:00–19:00 p.m.) through the WeChat official account of “YOUXUE UP.” The parents viewed through the official account, led their children to practice together at home according to the video requirements, and were required to record the practice process as a video and upload it. 4. After the researchers received the feedback, they commented and scored in a timely manner. The parents could ask questions through the “mutual evaluation” of the official account. 5. Parents who had not uploaded the tasks received task reminders.  6. According to the feedback, the researchers praised the children who completed all the tasks every week and provided small rewards as encouragement. | Empirical and theory | Research experts and educators developed the program, however program implementation relied on parents | Technology equipment needed (smartphone/iPad), parents and children needed to complete activity together | Yes | Yes | The main findings of this study suggests that this intervention effectively reduced the sedentary behaviour of of preschool children and their parents and increased light and moderate-vigorous physical activity, and the physical fitness (i.e. muscle strength, coordination, speed-agility, and balance) of preschool children. However, this intervention did not improve flexibility. Overall, this intervention was feasible and effective. |  |
| Kishida, K. | Youth | Unified Universal Prevention Program for Diverse Disorders (Up2-D2) program | The program consists of both cognitive behavioural and positive psychological interventions to address internalizing and externalizing problems and improve self-efficacy and social skills in children and adolescents. | 1. 12 sessions based on cognitive-behavioral and positive psychological interventions: psychoeducation about emotion (session 1), behavioral activation (session 2), social skills training (sessions 3 and 4), relaxation (session 5), strength work (session 6), cognitive restructuring (sessions 7 and 8), exposure (sessions 9 and 10), problem-solving (session 11), and review and conclusion (session 12). 2. Each session lasted 45 minutes and was conducted using the following procedures: (a) introduction (i.e., reviewing the last session and explaining the goal and purpose of the session); (b) learning target skills (i.e., cognitive-behavioral, or positive psychological skills); (c) practicing target skills (in both individual and group activities); and (d) conclusion (i.e., explaining the homework and summarizing the session). Worksheets were distributed in each session to assist the children in learning the program components.  3. Teachers were provided with teaching plans that included specific program procedures in school settings. | Empirical and theory | User-centred, training was implemented, educational and research experts were used | Although program implementation was found to be more effective with experts, trained schoolteachers were used | Yes | Yes | The results demonstrated that the intervention effectively reduced anxiety and to a greater extent for those with higher anxiety levels. This finding was maintained at a six-month follow-up. Students with higher anxiety showed improvements in internalizing and externalizing problems. However, there were no significant improvements for other mental health problems, self-efficacy, or positive attitude. |  |
| Lemes, V. B. | Children | Remote Physical Education Program | Physical education classes including movement and organized physical exercises were carried out by teachers through the online platform Google Classroom to improve and motivate daily physical activity as part as a routine during the pandemic. Parents and family were invited to join. | 1. The classes were organized by themes over 28 weeks. 2. All orientations were given to promote movements and organized physical exercises at home. It was explained and sent to parents and families an E-book with orientations about how to practice physical activity and physical exercises at home with a didactic, practical, and pedagogical approach. 3. The classes were developed and shared in google classroom on Monday. The teacher guided children and parents during the week about the movies, texts, and tasks. | Theory | Educators implemented and guided the program. Research expertise developed the program | Technology equipment needed (computer and network facilities) | Not reported | Yes | Although most children presented no individual changes in self-perceived physical activity, 20-27% of children increased in self-perceived activity. The online classes were concluded to be adequate for mainly children that were motivated to learn and practice physical education at home. |  |
| Lemos, G. | Children | Gulbenkian Academies for Knowledge (GAK) | GAK is a national network of 100 projects that are highly committed to the promotion and development of a set of social and emotional skills in children and young people under 25 years old: communication, resilience, critical thinking, problem solving, creative thinking, self-regulation, and adaptability. Each Academy is focused on promoting and developing one or more social and emotional skills through artistic, scientific, community, cultural or sports activities, in areas as diverse as education, health, social inclusion or technological. | Not reported | Empirical and theory | Not reported | Not reported | Not reported | Not reported | This study does not evaluate GAK as an intervention, but rather assesses the mental well-being of youth in the program. This study provides evidence on the relevance of promoting social and emotional skills during the pandemic. Half of the participants report that they had put into the practice strategies or activities learned/developed in the Academies and this was suggested to improve their emotional state. Activities most frequently reported were related to learning, self-knowledge, and self-regulation. |  |
| Levstek, M. | Youth | Virtual Music Sessions (but not an intervention per se) | Assessed 13 virtual music groups across three music education hubs, including ensembles that targeted young people with special educational needs, young people from lower socioeconomic status, and inclusive music production spaces. | Not reported | Theory | Not reported | Not reported | Not reported | Not reported | The main findings of this study found that young people showed a greater development in intra-personal outcomes and that relatedness support were indirectly related to virtual group music-making. Most relevant was that creative music-making and the community support by youth participation became a coping strategy for young participants while social distancing during the pandemic. |  |
| Li, L. | Children | The Adventures Aboard the S.S. GRIN program (Adventures) | This online intervention provided nine weeks of instruction through interactive episodes and a game-based environment to teach cognitive-behavioural strategies such as self-awareness, self-management, social awareness, relationship skills, and responsible decision making. | 1. The research team instructed teachers to implement Adventures for nine weeks, one episode per week, estimating 30–45 minutes per episode. Two additional weeks were added to allow teachers the opportunity to make up episodes with students as necessary, in total this was an 11-week program. 2. To prepare teachers to implement Adventures effectively, the Adventures development and research teams provided systematic training and supports to the teachers.  3. The research team used weekly digital newsletters, weekly online teacher implementation logs, phone calls, texts, and emails. The weekly log asked teachers to report their Adventures implementation format, session attendance, the content/episode covered that week, other intervention services students received, and whether teachers had questions or concerns about the implementation. | Empirical and theory | Training provided; research expertise used to develop program | Technology equipment needed (computer and internet) | Yes | Yes | Findings demonstrate that this intervention successfully improved social emotional skills. The main themes identified were that adolescent social media and technology use during school closures usually allowed for more and easier social connectivity, but the amount and relative ease of connectivity differed according to purpose and type of use. Emotions, particularly those of stress and happiness, were connected to whether adolescents actively or passively engaged with social media and technology. |  |
| Malboeuf‑Hurtubise, C. | Children | Online Art Therapy | The goal of this study was to compare a group-based and online emotion-based drawing intervention and a mandala drawing intervention. | 1. Sessions occurred through a secure, password protected, video conferencing platform, during which research assistants would log on remotely and join students in their classroom, using the classrooms’ smart board. 2. The emotion-based directed drawing intervention consisted of five weekly sessions during which children were instructed to complete varying drawing activities, that targeted exploring emotions and/or to provide an occasion to discuss. Students were asked to make drawings related to fear, worry, irritation, but also to draw how they were feeling. Pandemic-specific activities included drawing viruses and a cure to COVID-19. Group discussions followed each activity, during which children were invited to share their thoughts, emotions, and overall reactions to their drawings.  3. The mandala drawing intervention consisted of 5 different mandala drawing sessions taken from the CBT Art Activity Book. Following the drawing of their mandalas, children had the opportunity to discuss and share their reflections related to their drawings as a group. | Empirical and theory | Trained implementors, developed by research experts, evaluated with clinical expertise | Program leaders required training, technology equipment needed (internet, video conferencing platform, personal computer, or smart phone) | Yes | Yes | Findings demonstrate that both interventions were associated with mental health improvements, specifically inattention. Both an emotion-based intervention and a mandala intervention suggest being helpful in improving mental well-being for elementary students |  |
| Marques, S. | Youth | DISCOVER Workshop | This is a school-based one-day workshop for 16- to 18-year-old experiencing stress, anxiety, and/or low mood difficulties. The session covers stress management by teaching various cognitive behavioural therapy techniques (i.e., mindfulness and time management) to mitigate school-related stress, reduce worry and improve mood. | 1. A one day group workshop where students are taught cognitive behavioural techniques including: thought challenging, time management, and mindfulness 2. An individual psychology assessment is taken before the workshop and at three-months after. | Empirical and theory | Psychology expertise used for assessment; research experts developed program | Community venue required as it is delivered in-person | Yes | Yes | Many participants used the techniques from the DISCOVER workshops to cope with the lockdown. The techniques found to be most useful were those that helped reduce procrastination and improve time management. Relaxed breathing was also found to be helpful. Other popular techniques were mindfulness, sleep tips and strategies to challenge thoughts. |  |
| Özedemir, M. | Children | Psychoeducation program | An online six-session psychoeducation programme, based on the solution-oriented approach to improve problem solving skills and emotional resilience of primary school students. | Not reported | Empirical and theory | Not reported | Technology equipment needed, network and video platform issues arose | Yes | Not reported | The experimental group showed a positive difference between the pretest and post-test scores for problem-solving and emotional resilience and thus was shown to have a positive impact on children’s problem-solving and emotional resilience skills. |  |
| Paiva, V. | Youth | Online psychosocial research program combined with engagement in health promotion | An adaptative youth-agent research program during the pandemic that included weekly counselling sessions and youth led mental health promotion activities. | Activities in the program included:  1) Individual counselling through audio or written messages on WhatsApp were used when privacy issues did not allow for a live voice. Group meetings were held through other mobile applications (Zoom, Google Meet and Jitsi), given that most of the young adults do not have computers at home or stable internet access.  2) Youth agents (Yas) led mental health promotion. Live video streaming sessions were initiated and mediated by YAs through Facebook, and educational material was produced by the YAs, who organised its dissemination via WhatsApp and Instagram to their peers, with close supervision and support.  3) In impoverished neighbourhoods, strong youth involvement mobilised their own community-based organisations to foster social responses to SARS-CoV-2.  4) Young people actively produced and disseminated educational material, under our supervision. Students developed posters encouraging social distancing and continuing to stay connected and disseminated through social media.  5) Students organised and coordinated a Google-meeting live session. They had collected questions among their colleagues and invited researchers in our team as the ‘experts’ on the panel and encouraged critical reflection about the limits of individualising and medicalizing all mental distress. | Theory | Youth engagement, research expertise | Technology equipment needed | Not reported | Not reported | It was found that COVID-19 increased the burden of grief, fear, and anxiety. Often the teachers’ appreciation for having contact to enable dissemination of information about social and psychological support networks was often combined with requests for help. However, the youth-led initiatives developed over the course of the program demonstrated how students can encourage social connectivity without being in-person. Strong youth involvement mobilised their own community-based organisations and dissemination of education material to foster social responses during the pandemic. |  |
| Thompson, H. | Youth | The Bigger Picture campaign (TBP) | Youth participated in culturally relevant arts-based expression programming in a range of settings that both teaches and provides space to reflect on the social determinants of disease, including the structural, social, and environmental factors that influence exposure, behavior, and disease risk. Public health topics covered included type 2 diabetes, COVID-19, climate change, police brutality, and mental illness. | 1. Youth in 6 schools received virtual programming once per week over the course of six-week school-day residency programs. Staff partnered with classroom teachers within participating schools to deliver TBP/ Traditional content.    2. To augment programming for virtual learning and to facilitate peer-to-peer interactions, TBP/ Traditional both used break-out sessions to support small group interactions and relied heavily on the “chat” function, which enabled students to communicate directly with each other, even if their microphone/camera were off.  3. In traditional schools (control group), Poet Mentors led workshops on the formulation of voice and identity, often in response to the standard curriculum (e.g., using discussions, free-writes, and performances to allow students to think more deeply about their personal relationship to the themes and topics that came up in their English readings).  4. In TBP schools, workshops featured the same approach to voice, identity, and expression. However, rather than respond to the standard curriculum, the TBP curriculum was intentionally designed to increase interest in civic engagement and shift mind-sets and expectations by helping students recognize, understand, and engage with the structural factors that disproportionately impact the health of low-income communities and communities of color. | Empirical and theory | Educational leadership, youth staff, and wellness coordinators implemented intervention; research expertise used to develop program | Difficulties engaging youth virtually | Yes | Not reported | Students in this intervention reported an increase in students’ mind- sets around structural, social, and environmental determinants of health and health equity, as well as motivated plans for civic engagement. The integration of civic engagement opportunities into the curriculum suggested a positive impact on students’ reported desire to work for social change. |  |
| Waters, L. | Youth | SEARCH (Strengths, Emotional management, Attention and awareness, Relationships, Coping, and Habits) - Positive Education Intervention | A whole-school positive education intervention that focuses on six key pathways to well-being: Strengths, Emotional management, Attention and awareness, Relationships, Coping, and Habits and goals (SEARCH). Students are provided activities in class to build their skills in each goal. | 1. Teachers at the school were trained in the “SEARCH” pathways and given activities to run in classrooms that help students learn skill that allow them to build up the six pathways of strengths (e.g., strengths pathways: strength surveys and strengths challenges), managing their emotions (e.g., learning how to label the full spectrum of emotions and identifying emotions through a mood-meter), focusing their attention (e.g., mindfulness), building their relationships (e.g., active-constructive responding), coping (e.g., cognitive reframing and breathing techniques), and building habits and setting goals (e.g., if-then intentions).  2. Students rated the degree to which they had been taught these skills and how much they engaged in these coping strategies | Empirical and theory | Not reported | Not reported | Not reported | Not reported | Teaching positive education prior to COVID-19 had positive correlations with the way students coped during remote learning (positive reappraisal, emotional processing, and strengths use) and with stress-related growth when returning to campus. More specifically, the more that students reported they had been taught the skills for increasing their levels of “SEARCH” the more they were likely to utilize adaptive coping skills during remote learning. |  |
| Yuan, Y. | Youth | Mindfulness training (MT) | Adolescents identified with low resilience were invited to participate in a mindfulness training intervention. | 1. All the participants were instructed to listen to approximately 15 min of a mindfulness training recording every day for 6 months.  2. Each training task was sent to students by the teacher through Dingding, a homework software, to supervise the students’ daily training. The teacher set up a learning group for 90 students and sent mindfulness training recordings every night.  3. All subjects were required to check in to complete the homework every night.  4. Subjects were excluded if they missed at least one training task per week for two consecutive weeks. Additionally, to supervise each subject’s participation, a certain amount of credit was deducted as punishment for students who failed to complete the task. Subjects who adhered to mindfulness training at least six times per week received small prizes to promote subject retention. | Empirical and theory | Educational leadership implemented training; research expertise developed program | Not reported | Not reported | Not reported | Mindfulness training increased students’ emotional intelligence and resilience. There were significant individual differences in the initial level and development speed. When undergoing a mindfulness training intervention, individual emotional intelligence can promote the growth of resilience. To combat the generation of negative emotions towards the pandemic, authors recommend encouraging acceptance and mindfulness. |  |
| Zheng, Y. | Youth | Recess and Exercise Advocacy Program [REAP]- Digital behavior change intervention | The live-streaming app encouraged students to exercise and practice eye relaxation techniques as well as share videos of them participating in physical activity and engage with other classmates’ videos. | 1. The intervention group received a health information session, online curriculum, workout videos, and breaks. Students in the intervention group were asked to download the peer-to-peer live-streaming app REAP.  2. During each at-home recess (15 mins for each recess), participants in the intervention were prompted by SMS text messaging to log in to the REAP app and participate in live streaming and post their workouts.  3. With the help of their parents’ children could livestream their own workout videos/photos. After uploading, children could share motivational messages or hit the like button to increase their engagement with the program. The class administrators who were not involved in this study supervised and approved all the uploaded content. | Empirical and theory | Training required, manual of operations was provided, software engineer developed app, research expertise was used to develop program | Technology equipment needed (smartphone/iPad), parents needed to help post videos | Not reported | Not reported | This digital behavior change intervention reduced children’s anxiety and eye strain during COVID-19–associated online schooling. The reduction in self-reported eye strain and anxiety was significantly greater in the intervention group than the control group. |  |

^1^Children defined as 1-12 years of age. Youth defined as participants 13-18 years of age. Children and youth are populations that include both age groups.

^2^Brief description of the approach

^3^Steps or guiding principles to conduct the approach.

^4^Approach is derived from empirical evidence (i.e., through observation or experiment) or from published theory.

^5^Minimum expertise required to conduct the approach.

^6^Limitations to the approach

^7^Is the approach reproducible? (i.e., evidenced by use at multiple settings)

^8^Can the approach can be feasibly applied to other contexts? (N.B. decisions were made based on that internal validity should precede external validity)

^9^Relevant conclusions and recommendations identified that inform approaches to mitigate deleterious impacts of the COVID-19 pandemic on children and youth.

**Supplemental Table 4.** Summary of evidence suggested from included records to inform future approaches, strategies, to interventions to mitigate deleterious impacts on children and youth in future public health crises.

| **First author** | **Year** | **Outcomes** | **Population^1^** | **Author’s Conclusions and Recommendations** |
| --- | --- | --- | --- | --- |
| Arslan, G. | 2022 | Strength-based parenting, strength’s use, school belonging | Youth | Strength-based parenting had a significant predictive effect on academic motivation. School belonging was seen to have a mediating effect between strength-based parenting and academic motivation. Thus, finding ways for students to connect to school life, teaching young people to acknowledge and use their strengths, especially through parenting can be improve academic motivation during future pandemics and other adversities. |
| Bourion‑Bédès, S. | 2022 | Physical well-being, psychological well-being, school environment, health-related quality of life, social support, parental well-being | Youth | As a high level of parental anxiety was associated with children’s impaired health-related quality of life, identifying factors that lead to higher parental stress during the pandemic and developing interventions to decrease parents’ levels of anxiety will be beneficial to children and adolescents and improving their health-related quality of life. Physical exercise was perceived as a very effective means to relieve stress and served as a protective factor against impaired health-related quality of life. Thus authors recommended promoting physical activity among youth. Schools can support this by sending exercise plans for at-home physical activity. Further, a low level of quality of life was found with lower social support thus, facilitating social interactions is necessary. |
| Bryce, C. | 2022 | Hope, connectedness | Youth | Hope positively predicted feelings of school connectedness among middle-school and high-school students during the pandemic. Hope may be an important protective factor to overcome educational disruptions and increase motivation, especially as students begin to return to in-person learning. Supporting students' goals and improving hope may help increase their school motivation. Interventions that cultivate coping skills may be important to mitigate stress. These findings support consistent daily social activities, even if circumstances call for these activities to look different in an online format. |
| Cortés-García, L. | 2021 | Mental health, financial status, race, media, coping strategies during the pandemic | Children | Authors recommend leveraging community support; promoting social and emotional skills; providing health promotion sessions or materials at schools that reinforces familism and sense of belongingness to the community; providing structured scheduling for youth such as sessions to connect with peers face-to-face online or the inclusion of a social hour; provide youth and families with online physical activity programs that motivate them to have a healthy lifestyle at home; having a balanced diet and keeping regular sleep patterns; to measure screen time spent by children and adolescents during the pandemic and ensure responsible reporting of science in the media and its impact on mental health. |
| Gadermann, A. | 2020 | Optimism, life satisfaction, sadness, pandemic-related experiences | Children | After accounting for student demographics and prior well-being levels, relationships that improved with parents and older adults at home during the pandemic was suggested to be one of the most important predictors for students' optimism, life satisfaction, and lower levels of sadness. Spending time with family in-person and virtually had helped to cope with pandemic-related stress. Relationships with teachers and other adults were significantly associated with higher optimism and life satisfaction during the pandemic. Improved relationships with peers were also significantly associated with higher optimism. Thus, authors recommend prioritizing socializing safely. Further, as reflected by their top choices to deal with worries and stress, authors recommended promoting explored interest and outdoor activities. |
| Godawa, G. | 2020 | Personality trait, perceived stress, types of covid-19-related threats and coping strategies | Youth | More than a quarter of adolescents used planful problem solving as a coping strategy in a stressful situation or event. This was followed by escape-avoidance and seeking social support. Authors recommended: to give agency to older students plan activities and organize their social life, provide agency for student to work on tasks in a planned manner; offer a platform for youth to engage in social discussions; build self-education and self-development as positive responses to stressful situations and diminish escape avoidance strategies; expand and strengthen the network of relationships with loved ones; ensure young people will be able to use the media in a safe way and ensure reliable media education; involve youth in volunteering and other opportunities. |
| Grimes, A. | 2022 | Facilitators and barriers to physical activity, parent/student recommendations for increasing physical activity through sports/activities, facilitators and barriers to fruit and vegetable consumption, parent/student recommendations for increasing fruit and vegetable consumption | Youth | The most common motivation for physical activity were found to be peer influence, socialization, parent involvement, and enjoyment. The barriers for physical activity during the pandemic was time commitment and reduced motivation. To increase physical activity during the pandemic, students and parents suggested leveraging competition and setting goals. Wearable activity monitors for self-tracking, competition, and goal setting were also suggested.  Fruit and vegetable consumption were often managed by parents. Getting their children to eat healthy foods was found to be a challenge. Findings showed that though students knew the benefits of eating healthy but reported complaints about the preparation and freshness of fruits and vegetables offered at schools. Researchers recommend developing an intervention that is fun and engaging such as cooking at home. Parents also suggested cooking challenges as well as using social media to engage students. |
| Ho, T. | 2022 | Depression, stress, resilience, life satisfaction | Youth | Findings demonstrate students with high resilience are less affected by academic stress than students with low resilience. The impact from academic stress and resilience was seen as a greater effect with adolescents with low life satisfaction. High resilience and high life satisfaction were found to have the lowest risk of depression, in contrast to those with low resilience and low life satisfaction which had the highest risk of depression. Authors conclude that enhancing resilience and life satisfaction is important in preventing and managing depression in adolescents. |
| Jones, S. | 2022 | Connectedness, mental health, suicidal thoughts, and behaviours | Youth | Poor mental health outcomes were present across students of all sex, sexual identity, and racial and ethnic groups. However, prevalence of poor mental health and suicidality were less among students that felt close to persons at school and were virtually connected with others during the pandemic. Researchers view efforts to improve connectedness to schools, peers, and family as critical to protect the mental health and well-being of youths, especially during adverse circumstances such as the pandemic. Recommendations included comprehensive approaches that promote help-seeking behaviors, connections to trusted adults and supportive peers, and engagement in community activities. Tangible interventions discussed were schoolwide programs focused on social and emotional learning, professional development for staff to improve classroom management, and to analyze school policies to ensure they are being implemented equitably across racial and ethnic groups. Parents and caregivers can also build relationships with their child through open discussions and shared activities. |
| LaForge-MacKenzie, K. | 2022 | Participation in extracurricular activities and sports, mental health outcomes (depression, anxiety, hyperactivity, and inattention) | Youth | Students who participated in school sports during the first year of the pandemic reported better mental health and lower depressive symptoms compared with non-participants. Those who participated in sports prior to the pandemic showed lower inattention, lower anxiety, and hyperactivity compared to non-participants. Therefore, researchers established the impacts of the absence of school sports during the pandemic. |
| Liang, E. | 2022 | Interactions with social media and technology, social connectivity, mental well-being | Youth | Findings demonstrate that adolescent social media and technology use during school closures usually allowed for more and easier social connectivity and allowed relief for pandemic-related stressors. However, social media and technology also increased stress about current events and bullying due to increased screen time. Emotions, particularly those of stress and happiness, were connected to whether adolescents actively or passively engaged with social media and technology. Thus, researchers encouraged the development and dissemination of strategies guiding adolescents on how to personalize their social media to ensure positive experiences. |
| McCluskey, G. | 2021 | Students' concerns about mental health during the pandemic | Youth | This study found that students felt there was not enough support after schools reopened, especially for those more vulnerable. Students recommended more in-school mental health and learning supports from teachers, counsellors, and youth leaders, time for reflection after the experiences of the pandemic, and open conversations about the impacts of the pandemic. From their findings authors recommended; for staff to close the gaps caused by the loss of learning, improving mental health and well-being training for staff, ensuring structured opportunities for reflection, listening, and sharing of experiences, making school counselling as a norm, expanding opportunities for peer support, and promoting support beyond school. |
| McGuine, T. | 2022 | Sport participation, mental health, physical activity, health related quality of life | Youth | After controlling for grade, sex, school instructional delivery method, and the percentage of students qualifying for free or reduced-price lunch, findings demonstrated that athletes who returned to school and did not play sports in the fall of 2020 experienced worse symptoms of anxiety and depression, lower levels of physical activity, and worse health-related quality of life, than athletes who did play sports during that time. This suggests that the reinitiation of sport participation may provide significant improvements in mental and physical health for youth during the COVID-19 pandemic. Thus, it is recommended that physical activity should be promoted during future pandemics. |
| Oinas, S. | 2022 | Self-regulation of their learning environment, effort and peer learning, positive experiences of self-regulated learning and remote learning, worries and support needs | Youth | Findings from this study indicate that a positive remote learning experience was related to participation in peer learning and the ability to regulate their effort. Authors recommended teachers and technology developers to; develop self-regulation skills in students consistently, teach self-regulation skills tailored to the students' age and developmental stage, clearly lay out new skills and knowledge with guidelines, enhance peer learning opportunities, make online learning environments accessible and equitable for all students. |
| Parker, J. | 2021 | Coping strategies identified by black youth including religious and spiritual based support, emotion-focused coping, problem- focused coping, and social support | Youth | Youth that the most challenging experiences during the pandemic was a loss of normalcy due to a change in their routine, the limited social interactions, online learning, and mental health and trauma related experiences. Authors found the strategies most commonly used were personal coping, positive reappraisal, religious and spiritual coping, social and family support and school and religious community support. Therefore, authors recommended to provide culturally responsible services and that school-based mental health support should support Black students' use of religious and spiritual resources if they are desired. Recommendations to support Black students also included actively involving youth, offering opportunities to share their concerns, connecting families to community resources, and referring students to mental health professions if required. |
| Sciacca, B. | 2022 | Time spent online, children's self-regulation, children's digital skills reported by parents and their children | Youth | The two main strategies described when mediating children's media use was active and restrictive mediation. Parents who preferred active mediation actively discuss media content with their child, while parents that prefer restrictive mediation set rules with their children when using media. Findings showed that children whose parents applied higher levels of active and restrictive mediation reported higher digital skills, and they spent the lowest amount of time online when their parents employed higher levels of restrictive and lower levels of active mediation. In general, parents who adopted more mediation during lockdown were more likely to have more negative attitudes towards digital technology and lower digital skills, and to be more worried about online risks. Authors recommend parents consider a negotiated active and restrictive parental mediation strategy involving their children, which in turn is will likely protect children from exposure to online risks and foster their digital skills. |
| Silk, J. | 2022 | Daily emotional health, mood, daily depressive symptoms, anxious symptoms, activities engaged in during the day, negative impacts of the pandemic, positive impacts of the pandemic | Youth | Overall family relationships played an important role to adolescents' emotional health. Feeling less pressure from school, having more control over school, and having more free time for leisure activities were associated with reductions in negative mental health outcomes. The results from this study highlight the importance of providing in-person or quality online schooling, resources, and space for learning, promoting daily routines, and spending time with teens while reducing family conflict. Findings also recommend that finding ways to reduce school and achievement-related pressures and offering adolescent girls more agency and flexibility in managing their time is likely beneficial for their emotional health. |
| Soon, T. | 2023 | Adolescent coping strategies during the pandemic | Youth | Stress was found to be managed in three ways: disengagement by distracting themselves from their stress, taking active steps to cope with stress, and turning to others by using social support to help cope with social isolation and examination pressure. Authors recommend encouraging students to use adaptative coping strategies, to provide channels to check student understanding during lessons and to provide student opportunities to work together and interact. There must be a balance between maintaining academic rigour and being sensitive to student emotional states. |
| Wang, M. | 2021 | Coping strategies, parental support | Youth | Secondary control engagement coping (such as positive thinking, cognitive restructuring, acceptance, and distraction) was linked to same- and next-day increases in positive affect, indicating both immediate and sustained benefits. This coping strategy also moderated the link between health stress and negative affect. Parental support predicted increases in adolescents’ same- and next-day positive affect and decreases in same-day negative affect. Parental support also moderated the link between financial stress and negative affect. Thus, practitioners working to support youth during times of heightened health or financial stress may want to encourage the use of secondary control engagement coping strategies among youth and remind parents of how important it is for youth to feel their social and emotional support. |
| Zhu, Q. | 2022 | Peer victimization, resilience, quality of relationships, mental health difficulties | Youth | Peer victimization was found to be detrimental to students' mental health, especially among elementary school students. Resilience and positive teacher-student relationships buffered the impact of peer victimization and mental health challenges for elementary school students, but not secondary students. Findings demonstrated gender differences, as resilience predicted less mental health difficulties among elementary school boys, and positive teacher-student relationships predicted less mental health difficulties among secondary school girls. Researchers recommend that school psychologists and counselors conduct regular peer victimization and mental health screenings to design prevention and intervention programs, that transformative social emotional learning curriculums are implemented, that school staff are trained to recognize peer victimization, check in regularly and support to students, and that resilience is promoted by teaching students to identify their personal strengths, develop problem-solving skills, and practice positive thinking and emotional regulation skills. |
| Zhu et al. | 2021 | Emotional states, perceived vulnerability, impacts on lifestyle changes and social support | Youth | Findings found that more than half of the respondents reported no changes and those who reported favourable changes were higher than reporting unfavourable changes. Students were found to have better physical and mental health awareness and social support but reported decreased time spent for exercise. Additionally, students who reported higher perceived vulnerability also reported an increase in social supports. More female respondents reported negative emotions but were more likely to pay attention to their health, spend more time to relax and share their feelings. Male participants reported decreased social support after school closured. Primary and secondary school students did not differ much in negative impacts except that secondary school students reported more study stress and feeling more apprehensive. Generally, more primary school students paid increased attention to physical health and spent more time exercising, whereas more secondary school students spent increased time to rest and for relaxation. |
| Zhu et al. | 2021 | Health-related mindsets and expectations, sense of belonging, online learning, civic engagement | Youth | Although male students exhibited significantly higher game addiction behaviours, female students were more likely to report loneliness than male students, and those with excessive and pathological gaming behaviors reported more loneliness. The results showed that loneliness was positively associated with gaming addiction when adjusted for sociodemographic characteristics, but the association became negative when depression and anxiety were present. Home environments influenced patterns found in this study as students from single-parent families, with unemployed fathers, or with lower SES were more likely to exhibit gaming addiction behaviors. Authors recommend that policy makers must consider age and gender, and that parents and teachers with young children should encourage non-digital leisure activities to reduce the potential of frequent gaming. As alternative leisure options were limited during the pandemic, parental supervision is important. Authors also noted that if conducted for the right amount of time and in the appropriate contexts, gaming can be a healthy behavior. |

^1^Children defined as 1-12 years of age. Youth defined as participants 13-18 years of age. Children and youth are samples that include participants from both age groups.
